# A Texture-Hidden Anti-Counterfeiting QR Code and Authentication Method

**DOI:** 10.3390/s23020795

**Published:** 2023-01-10

**Authors:** Tianyu Wang, Hong Zheng, Changhui You, Jianping Ju

**Affiliations:** 1 School of Electronic Information, Wuhan University, Wuhan 430072, China; 2 School of Cyber Science and Engineering, Wuhan University, Wuhan 430072, China; 3 School of Artificial Intelligence, Hubei Business College, Wuhan 430079, China

**Keywords:** anti-counterfeiting QR code, Gaussian distribution, refined QR code, decoding, fourier domain

## Abstract

This paper designs a texture-hidden QR code to prevent the illegal copying of a QR code due to its lack of anti-counterfeiting ability. Combining random texture patterns and a refined QR code, the code is not only capable of regular coding but also has a strong anti-copying capability. Based on the proposed code, a quality assessment algorithm (MAF) and a dual feature detection algorithm (DFDA) are also proposed. The MAF is compared with several current algorithms without reference and achieves a 95% and 96% accuracy for blur type and blur degree, respectively. The DFDA is compared with various texture and corner methods and achieves an accuracy, precision, and recall of up to 100%, and also performs well on attacked datasets with reduction and cut. Experiments on self-built datasets show that the code designed in this paper has excellent feasibility and anti-counterfeiting performance.

## 1. Introduction

Anti-counterfeiting techniques can prevent or identify counterfeits to a certain extent, and traditional techniques can be classified into the following categories: visual anti-counterfeiting, electronic identification anti-counterfeiting, electronic code anti-counterfeiting, and texture anti-counterfeiting. Visual anti-counterfeiting mainly uses laser holography [1,2,3,4], special inks [5,6,7,8], temperature changes [9,10], security lines [11], chemical substances [12], and so on. They have special materials or unique formulations that not only cost a lot but also lose security once their information is leaked. Electronic identification anti-counterfeiting [13] commonly includes radio frequency, magnetic recording, integrated circuit cards, etc. These techniques work in conjunction with data management systems but they are not as generalizable as relying on specialized devices and are limited in application scenarios and scope. Based on the uniqueness of texture, all digital images of texture anti-counterfeiting marks [14,15] are obtained by high-definition photography, and then a database is established to upload, number, and save them. Consumers take photos with their mobile phones and retrieve the corresponding images saved in a database for human-eye comparison, but there is a lack of objective and intelligent means of identification. Traditional techniques suffer from various problems, such as excessive cost, poor user experience, and poor objectivity.

Different degrees of random toner adsorption occur in a digital graphic during printing, ensuring its physical non-replicability. The special design of the graphic can further amplify the distortion phenomenon, which can be identified by the algorithm. QR codes are widely used due to their strong coding ability and large information capacity. As QR codes are composed of a large number of black and white blocks, they are insensitive to the noise and distortion generated during printing. Therefore, the fusion of anti-counterfeiting graphics with QR codes is gaining increasing attention. At present, the fusion can be summarized as physical unclonable function (PUF) [16,17,18,19], watermark [20,21,22,23], copy detection pattern (CDP) [24,25,26,27,28], and halftone [29,30,31,32]. Although the above methods can achieve certain anti-counterfeiting effects, there are still some aspects that can be improved.

**Figure 1:**
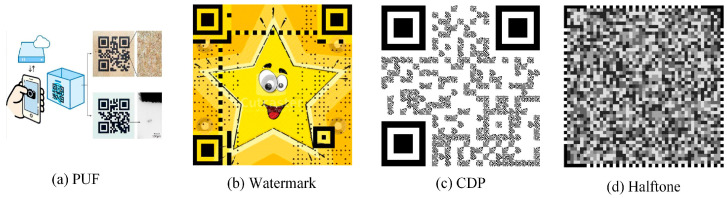
Anti-counterfeiting patterns related to QR codes.

For example, [16] utilizes natural texture features and printing micro-features, then calculates the feature similarity between the code to be tested and the sample code through the feature extraction algorithm; however, this requires high-precision printing and capturing equipment. The authors in [20] adopt the improved discrete wavelet transform and singular value decomposition algorithm to hide the digital watermark in the 2D code. In [21], the researchers embed specific random micro-textures into a 2D code and then convert them into a security layer. Any degradation process due to counterfeiting will change the statistics of the micro-textures and thus achieve anti-counterfeiting. However, watermarking algorithms can show some robustness to relatively low-intensity attacks, while for high-intensity attacks, the watermarking information is altered or significantly lost. A new 2LQR code is proposed in [24] to replace the black block of the QR code with a black and white module of the same size with anti-copy ability. This system includes public and private storage levels; the public level ensures that the decoding program can decode, and the private level uses the sensitivity of the module to the print capture process to distinguish real from fake codes. However, this code would attract visual errors, where it is easy for the naked eye to find the specific structure of the module, while also requiring precision in the capture device, which is not conducive to commercial generalization. The authors in [29] propose a two-dimensional code anti-replication scheme based on the spectral and spatial bar code channel model. Two sets of spectral and spatial features are extracted from the channel model and identified in a cascaded combination. However, the feature extraction process of this method is relatively complicated. In [30], the researchers add a double security authentication to the position of embedded information and then reduce the interval distance through fourth-order modulation. In this way, it is easier to create differences after printing and capturing, thus achieving the anti-replication effect. Figure 1 shows some representative anti-counterfeiting patterns.

To address the above issues, this paper designs a texture-hidden QR code, based on the codec mechanism of QR codes, using Gaussian distribution and information-hiding technology. The licensee generates a digital image of the code and then uses an officially authorized printer to legally print the authentic product and paste it on the goods or documents for circulation. The forger does not have access to the original code digital image, so the process involves scanning, reprinting, and pasting the fake product or document on top of the original. Users upload codes by taking photos on their mobile phones, which can be used to verify authenticity. The code has abundant texture details and specific frequency characteristics which improve the anti-replication capability while maintaining the generality of the QR codes. An efficient quality assessment algorithm is also proposed to address possible blur in the authentication processing. The proposed method describes the DFT low-frequency region of a code and computes and compares its magnitude and azimuthal features to determine the presence and degree of blur. Finally, this paper proposes a dual feature detection algorithm that includes both a decodability analysis and DFT spectral features. Specially designed anti-counterfeiting textures will show different diffusion patterns after printing. The details of fake codes will be glued together in large quantities, the code points will be destroyed, and the decoding will fail. To ensure accuracy, this paper calculates the spectral similarity between the tested code and the sample code and further compares the eigenvalues in four feature regions. The contributions of this paper are as follows.

(1) This paper designs a texture-hidden anti-counterfeiting QR code to solve the problem of the easily illegally copied QR code.
(2) An effective quality assessment algorithm is proposed to judge the type and degree of blur.
(3) The proposed dual-feature detection algorithm is shown to cope with different forgery means, capture devices, and attack scenarios.

The paper is structured as follows: Section 2 introduces the design process of anti-counterfeiting QR codes, Section 3 describes the quality assessment algorithm and the dual feature detection algorithm for the code, Section 4 presents the experimental effects in detail, and Section 5 concludes the paper.

## 2. The Design of Anti-Counterfeiting QR Codes

**Figure 2:**
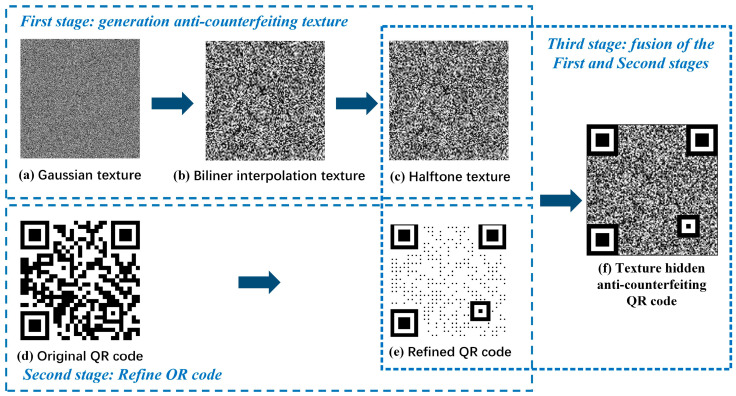
The generation process of a texture-hidden anti-counterfeiting QR code.

During the printing process of a digital pattern, the toner will be scattered randomly. Genuine codes only need to be printed once, while forged codes need to be printed at least twice, which can cause severe distortion. Realizing the above mechanism, this paper designs a texture-hidden QR code based on Gaussian distribution and information hiding to achieve an anti-copying effect. The code generation process is shown in Figure 2 and consists of three stages. The first stage is the generation process of an anti-counterfeiting texture. Random patterns generated from the Gaussian distribution function exhibit strong texture properties. Bilinear interpolation is used so that its gray level variation is moderately continuous, with more irregular gray level differences. Halftone operations are then performed by using error diffusion to bring the frequency of the digital code closer to the frequency sampled from the scanning and printing devices, and to improve signal aliasing during replication. The second stage is the refined process of the QR code. The semantic decoding process of QR codes first detects the position detection region and then detects the black and white code blocks. In this paper, the code points outside the position detection region and the calibration region are reduced, which does not affect the decoding. The third stage is the fusion of the above specially designed texture and the refined QR code at the code points. The anti-counterfeit code visually implements the information hiding of code points and has specific frequency characteristics, thus improving the anti-copy capability while maintaining the commonality of the QR code.

Step 1: Generate Gaussian random textures

**Figure 3:**
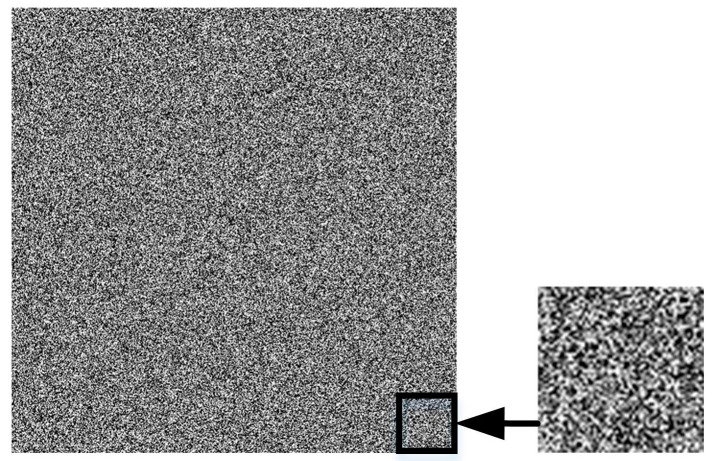
Gaussian random texture and its enlarged detail.

A random matrix is built from a Gaussian random generation function and visualized as a fine random texture pattern. The pattern and its enlarged detail are shown in Figure 3. Random patterns have the characteristics of a strong texture, and at the same time, have differences in each part, which increase the difficulty of counterfeiting. If the random variable $g_{t}$ follows a Gaussian distribution with expected value μ and standard deviation σ, its probability density function is:

(1) $$\begin{array}{c} g_{t}=\frac{1}{\sigma \sqrt{2\pi }}e^{-\frac{{\left(t-\mu \right)}^{2}}{2\sigma^{2}}} \end{array}$$

where μ determines the location of the distribution; σ determines the magnitude of the distribution; and $g_{t}$ is reconstructed into the matrix $G_{\left(x,y\right)}$.

Step 2: Use bilinear interpolation operation

**Figure 4:**
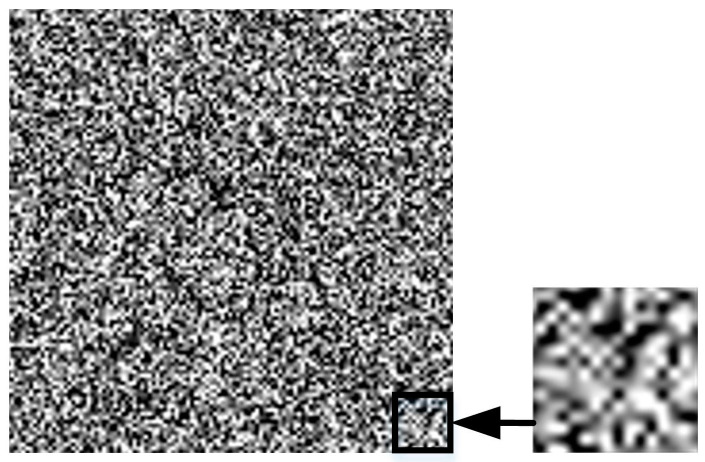
Bilinear interpolated texture and its enlarged detail.

Bilinear interpolation is to perform linear interpolation in both directions separately. Interpolation has the effect of low-pass filtering which can be anti-aliasing and effectively reduce some of the visual distortions caused by image scaling. Figure 4 shows the bilinear interpolated texture pattern and its enlarged detail. It can be seen that continuously varying grayscale levels are formed between pixels in the image details, with smooth patterns and increased low-frequency information. Bilinear interpolation takes the distance to the last four pixels as a reference weight and computes the integrated score by linearly interpolating it twice to obtain the pixel value of the current point. It can be expressed as follows:

(2) $$\begin{array}{c} W_{\left(x,y\right)}\approx \frac{y_{2}-y}{y_{2}-y_{1}}*R_{1}+\frac{y-y_{1}}{y_{2}-y_{1}}*R_{2} \end{array}$$

(3) $$\begin{array}{c} R_{1}\approx \frac{x_{2}-x}{x_{2}-x_{1}}P\left(G_{\left(x_{1},y_{1}\right)}\right)+\frac{x-x_{1}}{x_{2}-x_{1}}P\left(G_{\left(x_{2},y_{1}\right)}\right) \end{array}$$

(4) $$\begin{array}{c} R_{2}\approx \frac{x_{2}-x}{x_{2}-x_{1}}P\left(G_{\left(x_{1},y_{2}\right)}\right)+\frac{x-x_{1}}{x_{2}-x_{1}}P\left(G_{\left(x_{2},y_{2}\right)}\right) \end{array}$$

where $G_{\left(x_{1},y_{1}\right)}$, $G_{\left(x_{2},y_{1}\right)}$, $G_{\left(x_{1},y_{2}\right)}$, and $G_{\left(x_{2},y_{2}\right)}$ are the known points, and $P\left(G_{\left(x_{1},y_{1}\right)}\right)$, $P\left(G_{\left(x_{2},y_{1}\right)}\right)$, $P\left(G_{\left(x_{1},y_{2}\right)}\right)$, and $P\left(G_{\left(x_{2},y_{2}\right)}\right)$ are values of proportion; the closer points are more important.

Step 3: Adopt halftone treatment

**Figure 5:**
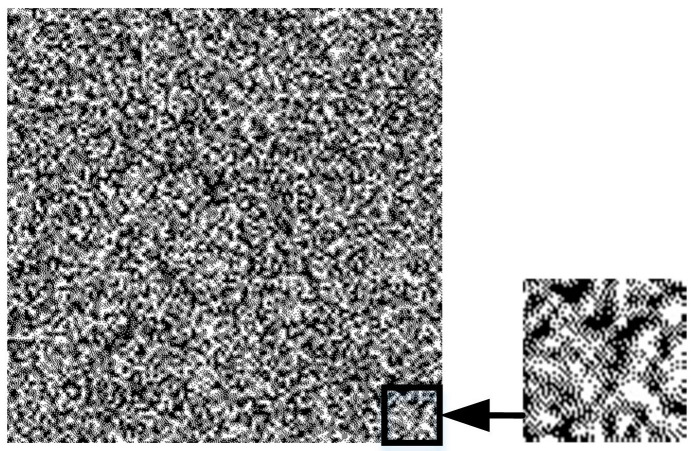
Halftone texture and its enlarged detail.

Since the printing device can only print black and white, the continuous gray tone image must be processed into a binary halftone image in the printing process, and the unit coverage area of small black and white dots that human eyes cannot distinguish is used to simulate the gray level change of the image so that the binary image looks as close as possible to the original image. In this paper, the error diffusion method proposed by [33] is adopted for the halftone processing of texture images, that is, every pixel in the image and its neighboring pixels are processed, and the error (the difference between the actual output and the original image) generated on a pixel is dispersed to the surrounding pixels in a certain proportion. After the above operation, $H_{\left(x,y\right)}$ is obtained. Figure 5 shows the texture pattern and its enlarged detail after the halftone treatment.

Step 4: Refine QR code

**Figure 6:**
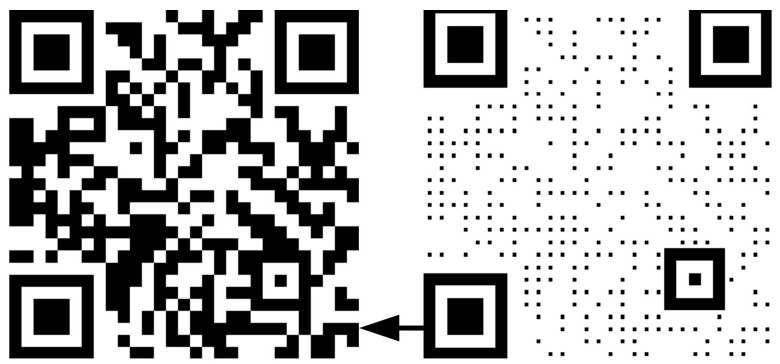
Original QR code and its refined QR code.

Figure 6 shows the QR code generated based on the Zxing open-source code and its refined code. First, the horizontal pixel values of the location detection area of the QR code are calculated and divided by the corresponding ratio (1:1:3:1:1) to obtain the minimum component module size. Then, the position and size of the calibration pattern are calculated according to the ratio characteristics of 1:1:1:1:1. Finally, the areas of the QR code are refined, except for the location detection area and calibration pattern, as follows:

(5) $$\begin{array}{c} RE_{\left(x,y\right)}=\left\{\begin{array}{cc} R(QR_{\left(x,y\right)}), & QR\;is\;data\\ QR_{\left(x,y\right)}, & Others \end{array}\right. \end{array}$$

(6) $$\begin{array}{c} R(QR_{\left(x,y\right)})=\left\{\begin{array}{ll} QR_{\left(x-a:x+a,y-a:y+a\right)}, & QR\;is\;the\;local\;center\;area\\ 255, & Others \end{array}\right. \end{array}$$

where $QR_{\left(x,y\right)}$ represents the value of the original QR code and a+1 is the reduced local pixel size.

Step 5: Combine texture patterns and refined QR code

**Figure 7:**
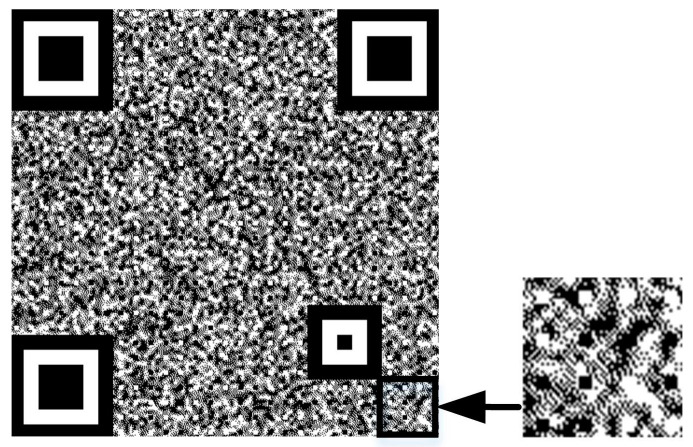
Anti-counterfeiting QR code and its local enlarged detail.

Figure 7 shows the combined anti-counterfeiting texture code and its details. The code points in the code data region are minor and nearly integrated into the anti-counterfeiting background. Since the position detection area is complete and the original QR code has a maximum error correction capability of 30%, the semantics of the anti-counterfeiting code can still be decoded by the code scanning device. The position detection area, calibration area, and black and white code points of the refined QR code are unchanged, while the rest of the code is replaced with Gaussian random texture patterns. The formula for this is as follows:

(7) $$\begin{array}{c} C_{\left(x,y\right)}=\left\{\begin{array}{cc} RE_{\left(x,y\right)}, & RE_{\left(x,y\right)}\;is\;data\\ H_{\left(x,y\right)}, & Others \end{array}\right. \end{array}$$

## 3. The Proposed Authenticity Approach

### 3.1. The Capturing Process of Anti-Counterfeiting Codes

**Figure 8:**
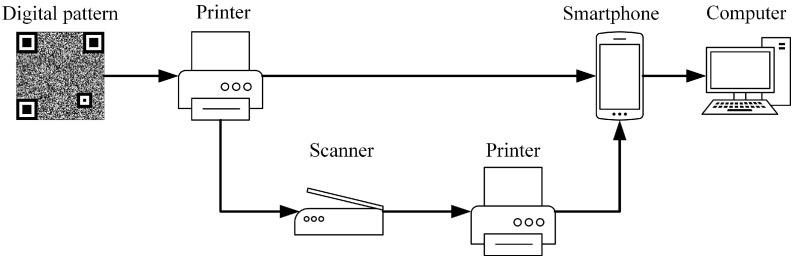
The capturing process of anti-counterfeiting codes.

Figure 8 shows the capturing process of anti-counterfeiting codes. Anti-counterfeiting codes are generated, printed, and attached to product packages by manufacturers. Counterfeiters do not have access to the original digital image and can only scan a high-quality printed code, which is then printed a second time after a series of image treatments. Consumers scan anti-counterfeiting codes through their mobile phones and other devices which then authenticates them.

**Figure 9:**
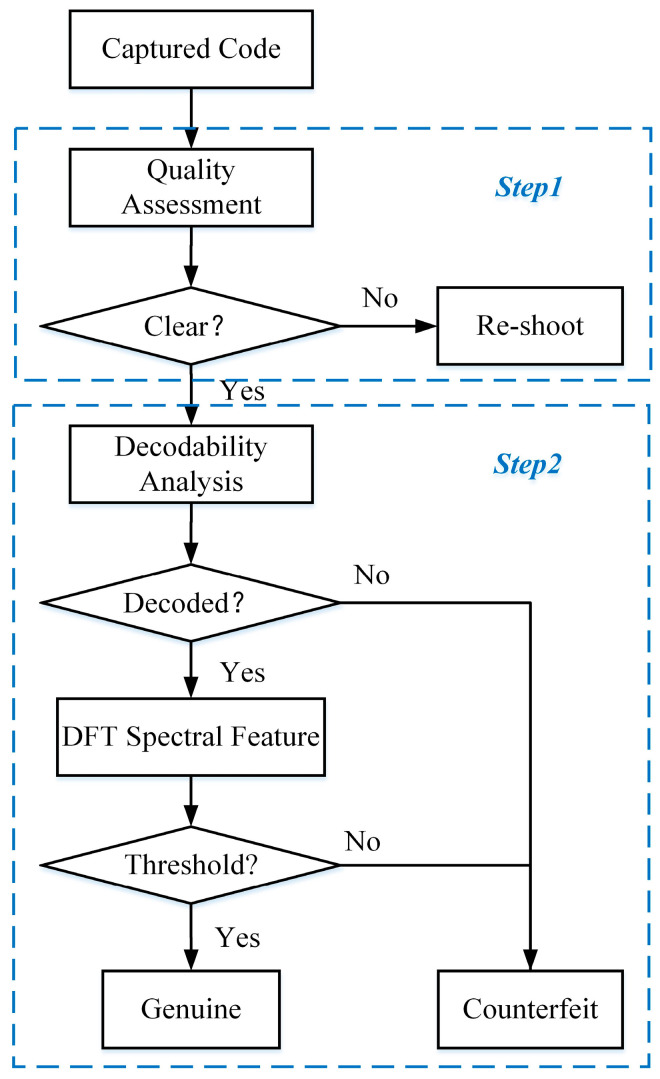
The authentication process of an anti-counterfeiting code.

As shown in Figure 9, the authentication process of the proposed anti-counterfeiting code consists of a quality assessment and dual-feature authentication. In the first stage, the image quality of the anti-counterfeiting code needs to be evaluated. Only anti-counterfeiting codes that meet the sharpness criteria will be tested. If they do not meet the criteria, the consumer is prompted to re-shoot. The second stage consists of a decodability analysis and DFT spectral feature detection. The former identifies whether the anti-counterfeiting code to be tested can be deciphered. If it cannot be decoded, it is directly judged as a fake. If it can be decoded, it is sent to the latter, which uses the decoded QR code semantic as an index to find the corresponding sample code in the sample database and compares it to compute the DFT spectral properties. If the similarity is greater than a threshold, it is judged as a fake, otherwise, it is judged as a genuine product. The combination of decodability and spectral features will improve detection efficiency while ensuring accuracy.

### 3.2. Quality Assessment Algorithm

The captured anti-counterfeiting codes may be blurred due to various factors such as environment, equipment, habit, etc. which typically manifest as defocus blur and motion blur. A clear code is particularly crucial for subsequent authentication. This paper transforms the code in the DFT frequency domain and calculates the magnitude and azimuthal characteristics of the low-frequency region to determine the type and degree of blur. The defocus blur is due to the incomplete coincidence of the image plane with the detector’s receptive plane, resulting in the lens not being clustered. Motion blur is caused by the relative motion between the image and the camera that captured it. The texture and edges of the image will become blurred, and its general expression is:

(8) $$\begin{array}{c} q\left(x,y\right)=p\left(x,y\right)⊗h\left(x,y\right)+n\left(x,y\right) \end{array}$$

where $q\left(x,y\right)$ is the output image; $p\left(x,y\right)$ is the input image; $h\left(x,y\right)$ is the point spread function; $n\left(x,y\right)$ is the noise; and ⊗ is the convolution operation.

The main difference between the two blurs is $h\left(x,y\right)$, in defocus blur, $h\left(x,y\right)$ is symmetrically distributed about the center of the circle, and in motion blur, $h\left(x,y\right)$ is asymmetric and related to both the angle and the length of the motion.

For defocus blur, $h\left(x,y\right)$ can be expressed as:

(9) $$\begin{array}{c} h\left(x,y\right)=\left\{\begin{array}{rr} \frac{4}{\pi r^{2}}, & x^{2}+y^{2}\leq \frac{r^{2}}{4}\\ 0, & others\;\; \end{array}\right. \end{array}$$

where r is the radius of the blur spot.

For motion blur, $h\left(x,y\right)$ can be expressed as:

(10) $$h\left(x,y\right)=R\left(\theta \right)+D$$

where $R\left(\theta \right)$ is a rotation matrix, which represents the rotation angle, and D is the displacement of the movement.

The proposed anti-counterfeiting code is characterized by slow and continuous grayscale variations between pixels, as well as rich low-frequency information. Therefore, it is used to evaluate the quality of the anti-counterfeiting code based on the DFT spectral image features. It can be translated into a frequency domain operation and can be written, regardless of noise, as:

(11) $$Q\left(u,v\right)=P\left(u,v\right)⊗H\left(u,v\right)$$

where $P\left(u,v\right),Q\left(u,v\right)$, and $H\left(u,v\right)$ are the 2D DFT transforms of $p\left(x,y\right),q\left(x,y\right)$, and $h\left(x,y\right)$, respectively.

**Figure 10:**
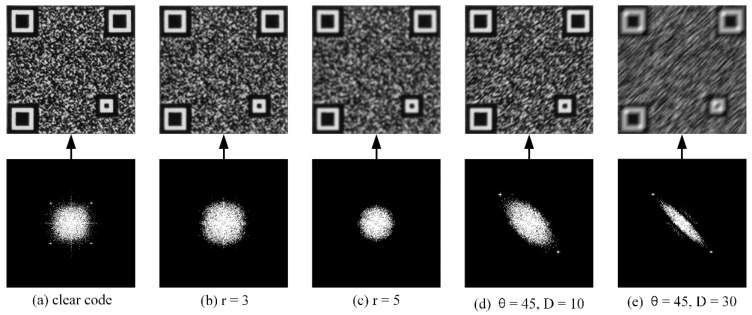
A clear code, four blurred codes, and their DFT spectrograms ((**a**) is the clear code; (**b**) and (**c**) are the defocused blur codes with a radius of 3 and 5, respectively; and (**d**) and (**e**) are the motion blur codes with displacements of 10 and 30, respectively).

**Figure 11:**
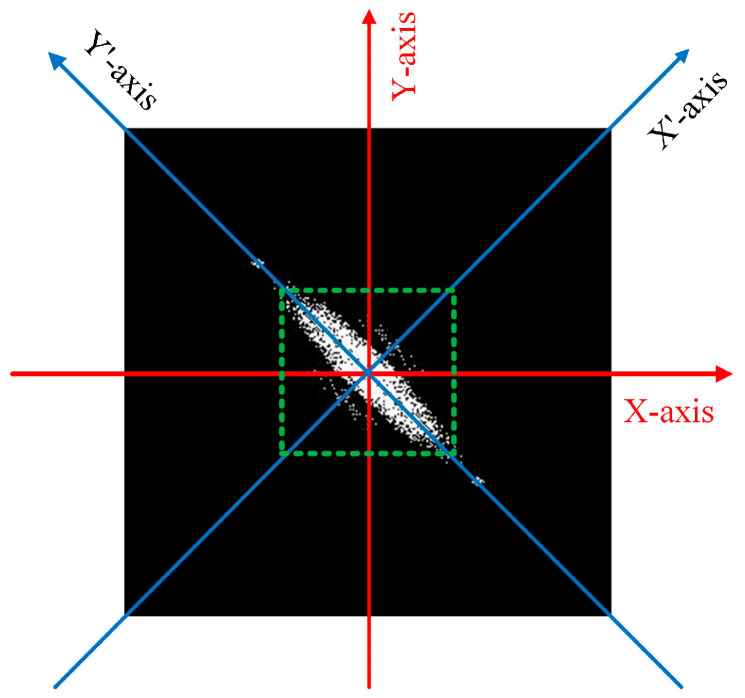
DFT spectrum of an anti-counterfeiting code.

Figure 10 shows one clear and four blurred codes, including their DFT spectrograms. It is obvious that the low-frequency region of a clear code is concentrated in an approximate rectangle. For defocus blur codes, the larger the blur radius, the fainter it appears to the naked eye, the more regular the circle formed by the low-frequency region of its Fourier spectrum, and the smaller the spectrum radius. For motion blur codes, the low-frequency region of the spectrum has an elliptical fringe with a fringe width inversely proportional to the displacement length and a fringe direction perpendicular to the displacement direction. Based on the blurred images above, it can be seen that the shape of the low-frequency region is strongly characterized by its magnitude and azimuthal features, as shown in Figure 11. The quality assessment algorithm, MAF, in this paper is described in detail below:

Step 1: Transform to the DFT domain and calculate low-frequency values in four directions. (1) Perform DFT frequency domain transformation on the anti-counterfeiting code, (2) move the DC component to the center of the spectrum, (3) calculate the normalization of the spectrum amplitude, (4) perform threshold process to obtain a binarized spectrogram, and (5) calculate $C_{X},C_{Y},C_{X^{\prime }}$, and $C_{Y^{\prime }}$ (white areas in the spectrogram) in the four directions of *X-axis*, *Y-axis*, *X’-axis*, and *Y’-axis*.

Step 2: Determine how blurred the image is and calculate the quality score of the code according to the following formula:

(12) MAF=MF+AF

(13) $$MF=\frac{C_{X}+C_{Y}+C_{X^{\prime }}+C_{Y^{\prime }}}{4}$$

(14) $$AF=\min\left\{C_{X},C_{Y},C_{X^{\prime }},C_{Y^{\prime }}\right\}$$

where MF and AF describe the magnitude feature and the azimuthal feature of the low-frequency region of the anti-counterfeiting code, respectively. The MAF is the quality score which is proportional to the sharpness of the code. If the score is greater than the threshold k, the code is determined to be clear, otherwise, the code is determined to be blurred.

Step 3: Determine the type of blurred code by using the following relation:

(15) $$pro1=\frac{C_{X}}{C_{Y}}$$

(16) $$pro2=\frac{C_{X^{\prime }}}{C_{Y^{\prime }}}$$

where pro1 and pro2 are the ratios of low-frequency regions on both axes, and the type of blur can be judged based on the values of pro1 and pro2. The dif is the error value; if pro1>1−dif and pro2>1−dif, the code is a defocused blur, otherwise the code is a motion blur.

### 3.3. Dual Feature Detection Algorithm

#### 3.3.1. Decodability Analysis

In this paper, random texture patterns are combined with refined QR codes to generate anti-counterfeiting codes. Due to the integration of QR codes, anti-counterfeiting codes do not require other special position symbols or marked information and can be directly decoded by a decoding program.

Printing or capturing comes with channel distortion and noise, and the decodability varies after the code is passed through different channels. For genuine codes that have been printed and captured only once, the resulting distortion and noise are small, and codes can still be recognized by the decoding device. For illegal channels that undergo multiple replicas, more severe distortions and noise are generated, with obvious forgery traces, and substantial texture details are lost. Stochastic diffusion phenomena exhibit different diffusion patterns, which can cause decoding to fail. Therefore, the decodability of the code to be tested can be used as an evaluation metric, and those codes that cannot be decoded are identified as fakes.

**Figure 12:**
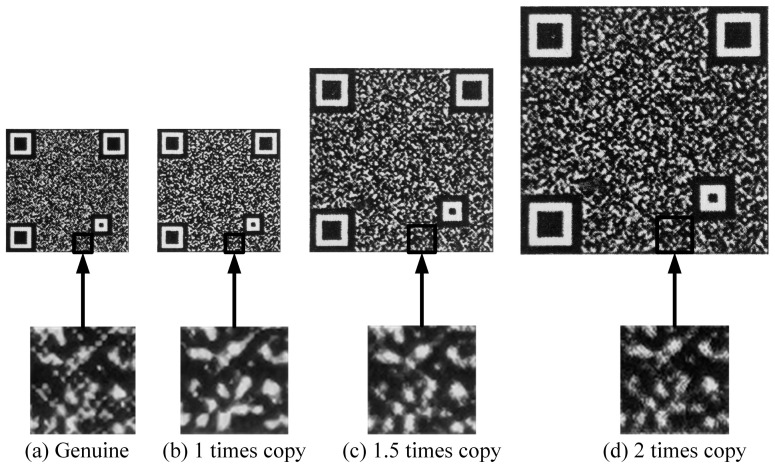
Genuine code and counterfeit codes in different scales and their enlarged details ((**a**) is the genuine code and its enlarged detail; (**b**–**d**) are the counterfeit codes in different scales and their enlarged details).

In the actual process of counterfeiting, the counterfeiters resort to superior-quality enlarged copies. On the one hand, consumers generally do not know the size of the code. The original size of the code is generally small, and a copy enlarged within two times will not make a particularly noticeable difference. On the other hand, product packaging includes not only a single outer package but also a boxed package. The use of enlarged anti-counterfeiting codes on the whole box packaging is not against the law and will deceive consumers. As shown in Figure 12, it can be seen that the texture details of the genuine code are rich, while the counterfeit code is glued and cannot be decoded by a code decoding device and can therefore be directly identified as a fake. The enlarged copies reconstruct texture details better than the equal copy, part of the codes can be decoded, and the first-level feature fails. Therefore, to ensure accuracy, it is also necessary to send the decoded code to the second-level detection, the DFT spectral feature.

#### 3.3.2. DFT Spectral Feature

The code can be regarded as a 2D discrete signal. The Discrete Fourier Transform (DFT) decomposes the signal into several sinusoidal plane waves and converts the code from the spatial domain to the frequency domain. It can be represented by the following equation:

(17) $$F\left(u,v\right)=\overset{M-1}{\underset{x=0}{\sum }}\overset{N-1}{\underset{y=0}{\sum }}f\left(x,y\right)e^{-j2\pi \left(\frac{u}{M}x+\frac{v}{N}y\right)}$$

where x and y are values of the space domain; u and v are values of the frequency domain, u takes 0 to *M* − 1, v takes 0 to *N* − 1; and $F\left(u,v\right)$ is the transform coefficients of $f\left(x,y\right)$. $F\left(u,v\right)$ can also be expressed as:

(18) $$F\left(u,v\right)=\left|F\left(u,v\right)\right|e^{j\emptyset \left(u,v\right)}$$

(19) $$\emptyset \left(u,v\right)=arctan\left[\frac{I\left(u,v\right)}{R\left(u,v\right)}\right]$$

(20) $$\left|F\left(u,v\right)\right|={\left[R^{2}\left(u,v\right)+I^{2}\left(u,v\right)\right]}^{\frac{1}{2}}$$

where $\emptyset \left(u,v\right)$ is the transformed phase angle; $R\left(u,v\right)$ and $I\left(u,v\right)$ denote, respectively, real and imaginary parts of $F\left(u,v\right)$; $F\left(u,v\right)$ is periodic and symmetric, which states that the Fourier spectral magnitude is concentrated at the origin:

(21) $$F\left(u,v\right)=F\left(u+A,v\right)=F\left(u,v+B\right)=F\left(u+A,v+B\right)$$

(22) $$\left|F\left(u,v\right)\right|=\left|F\left(-u,-v\right)\right|$$

**Figure 13:**
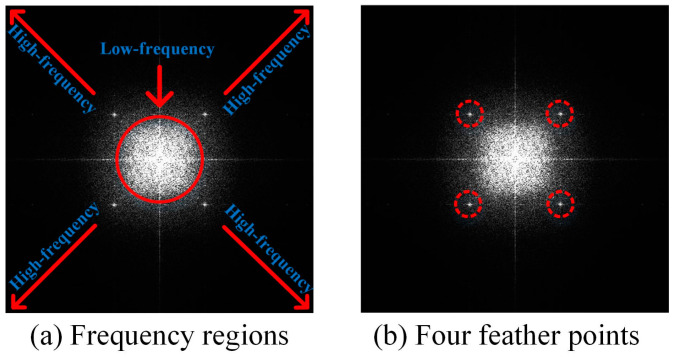
Spectrograms of a genuine anti-counterfeiting code.

**Figure 14:**
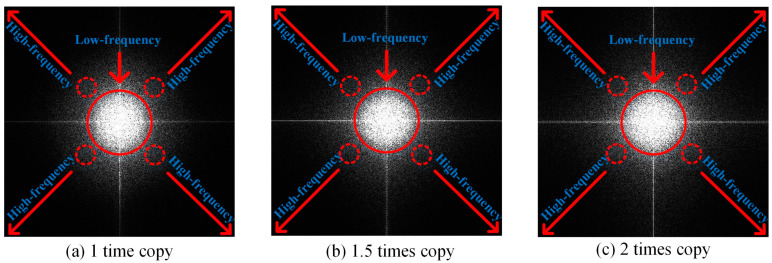
Spectrograms of counterfeit codes at different scales.

The frequency of an image represents the grayscale gradient, which represents the difference between a point and its neighborhood on a grayscale image. Figure 13 shows the spectrogram of a genuine anti-counterfeiting code and Figure 14 shows the spectrograms of the counterfeit codes at different scales. Regions where the grayscale varies gradually are represented by low-frequency coefficients and mainly include contours. The region where the grayscale changes sharply are represented by high-frequency coefficients, which mainly include noise and edges. Important feature points of the genuine code are clear and symmetric, but the points of the counterfeit code essentially vanish.

In this paper, the macro structure information and micro structure information of the code are extracted for authentication. The Normalized Correlation (*NC*) can be used to measure the overall spectral similarity between the code to be tested T and the corresponding sample code to be obtained S to yield the score of the objective evaluation, the formula for which is:

(23) $$NC=\frac{\sum_{x=1}^{M}\sum_{y=1}^{N}T_{\left(x,y\right)}*S_{\left(x,y\right)}}{\sqrt{\sum_{x=1}^{M}\sum_{y=1}^{N}T_{\left(x,y\right)}*T_{\left(x,y\right)}}*\sqrt{\sum_{x=1}^{M}\sum_{y=1}^{N}S_{\left(x,y\right)}*S_{\left(x,y\right)}}}$$

where M and N are the row and column of the spectrogram; the NC is between 0 and 1.

A larger NC indicates a higher similarity between two images. However, the overall features of some counterfeit codes are not significantly different from the genuine ones, but the differences in local features are more pronounced. Thus, this paper proposes the Pixel Ratio (PR) to extract four characteristic regions from the spectrogram. The higher the proportion of low-frequency in the characteristic region, the more likely it is to be a genuine code. The PR can describe this feature according to the following formula:

(24) $$PR=\frac{\sum_{x=1}^{d}\sum_{y=1}^{d}C_{w}\left(x,y\right)}{4d^{2}*256}\;w=1,2,3,4$$

where w is the *w*-th characteristic region; $C_{w}\left(x,y\right)$ is the pixel value of the *w*-th characteristic region, which ranges from 0 to 255; and d is the pixel length of a characteristic region; the PR is between 0 and 1.

Authentication can be achieved most effectively if the two metrics (NC and PR) are combined, here named NC_PR. Only if NC≥k1 and PR≥k2 is the code judged to be a genuine code, otherwise it is judged to be a counterfeit code. Here, both k1 and k2 are thresholds and can be selected to have values in a certain range.

## 4. Experimental Results

### 4.1. The Design Parameters of Anti-Counterfeiting Codes

The design parameters of the anti-counterfeiting code include the size of the code points, the expectation value of the random distribution, and the size of the physical print. Proper parameters can make the decoding of the counterfeit code fail to the greatest extent, provided that the decoding of the genuine code is guaranteed. This paper experimentally tests the effect of different parameters on the decoding performance and finally selects the parameters.

#### 4.1.1. Size of Code Points

**Figure 15:**
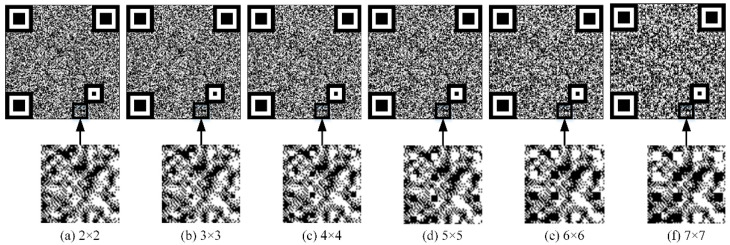
Anti-counterfeiting codes with different code point sizes and their enlarged details.

After selecting version number 3 and error correction level H to generate the original QR code, the modules of the QR code need to be refined to code points. The larger the code points, the more visual it is, the faster the decoding, and the smaller the area of the texture pattern. Thus, it is necessary to choose an appropriate code point size. Figure 15 shows anti-counterfeiting codes with different code points sizes of 2 × 2, 3 × 3, 4 × 4, 5 × 5, 6 × 6, and 7 × 7 and their enlarged details.

**Figure 16:**
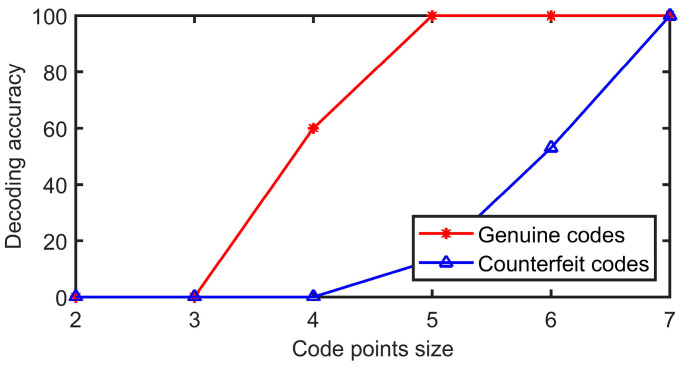
Decoding accuracy of codes in different code point sizes.

Five genuine codes with code points sizes of 2 × 2, 3 × 3, 4 × 4, 5 × 5, 6 × 6, and 7 × 7 are generated, among which, the expectation of Gaussian distribution is selected as 120, the physical print size is 1.2 cm, and the pixel size is 580 × 580. After printing, the same printer is used for copying and enlarging to obtain 15 counterfeit codes, for a total of 30 genuine codes and 90 counterfeit codes in six groups. The decoding experiment is shown in Figure 16. It can be seen that when the pixel sizes of the code points are 2 × 2, 3 × 3, and 4 × 4, the genuine codes cannot be decoded 100%, so they cannot be applied to the anti-counterfeiting code. When the pixel sizes of the code points are 5 × 5, 6 × 6, and 7 × 7, all genuine codes can be fully decoded and the decoding rates of the fake codes are 13%, 53%, and 100%, respectively. Therefore, considering the comprehensive guarantee of the decoding robustness of genuine codes and the decoding fragility of counterfeit codes, the size of code points is selected as 5 × 5 pixels.

#### 4.1.2. Expectation of Gaussian Distribution

**Figure 17:**
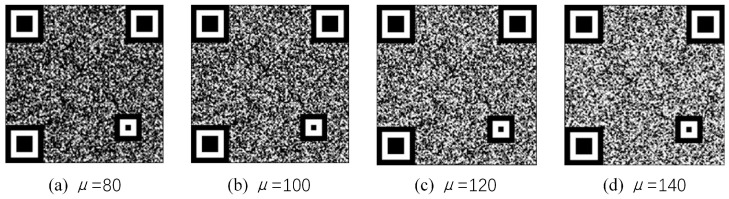
Anti-counterfeiting codes with different *μ*.

In the generation stage of anti-counterfeiting texture patterns, a random distribution function is used to generate detailed random texture patterns. The different Gaussian random distribution expectation (μ) determines the mean level of the pixel value of the texture patterns, which affects the visual effect of texture patterns and the decoding performance after combining with the QR code. The higher the μ, the higher the mean of pixels and the whiter the visual appearance; the lower the μ, the lower the mean of pixels and the darker the pattern tone. Figure 17 shows the codes with a μ of 80, 100, 120, and 140.

**Figure 18:**
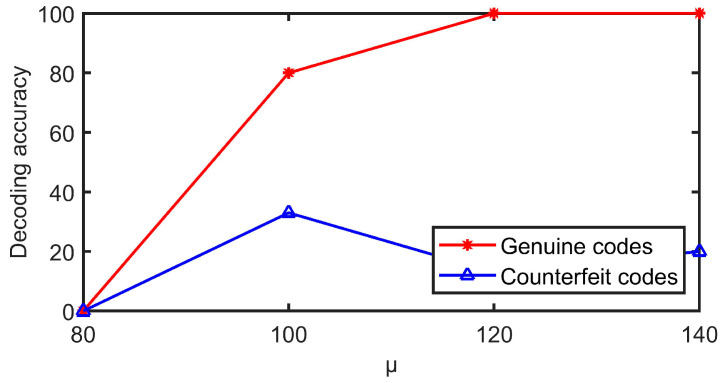
Decoding accuracy of codes with different μ values.

Five codes with Gaussian expectations of 80, 100, 120, and 140 are generated. Among them, the QR code version and error correction level are 3 and H, the code point size is 5 × 5, the print size is 1.2 cm, and the pixel size is 580 × 580. After printing, the same printer was used to copy and enlarge the copy to obtain 15 fake codes. In total, there are 20 authentic and 60 counterfeit codes. The decoding experiments were then performed and the results are shown in Figure 18. As can be seen, only the *μ* value of 120 and above can guarantee the decoding rate of the genuine code is 100%. When the *μ* value is 120, the decoding rate of the fake code is 13%, when the mu value is 140, the decoding rate of the fake code is 20%. At this time, the image field of view is white, the code points are obvious, and the code points are not well hidden. Therefore, in order to consider the visual hiding effect of code points and ensure the gap between the genuine and fake decoding rate, the μ is selected as 120 in this paper.

#### 4.1.3. Size of Physical Print

**Figure 19:**
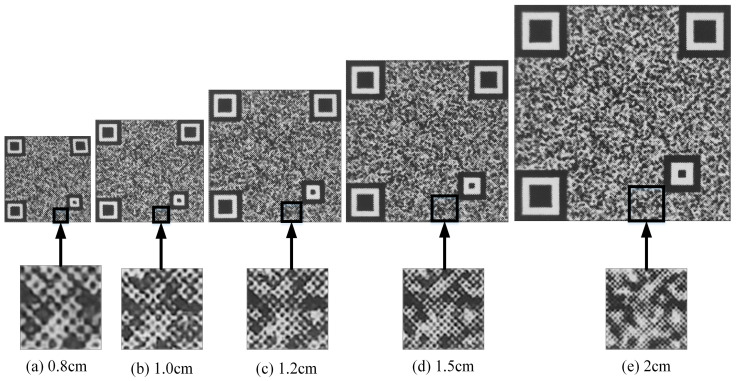
Anti-counterfeiting codes with different sizes of physical print and their enlarged details.

After the anti-counterfeiting code is generated, the code is printed and made into a label. Different print sizes can affect the decoding performance of the code. The larger the physical print size, the more complete the details and the easier the code to decode. Similarly, counterfeit codes that have been illegally copied can be easily decoded. Figure 19 shows the codes and their enlarged details for the print sizes 0.8 cm, 1 cm, 1.2 cm, 1.5 cm, and 2 cm. DPI is an important parameter that describes the printing accuracy of a printer. The higher the DPI, the more points are displayed within an inch, and the higher the printing accuracy. Current market circulation printers are generally 600 DPI or 1200 DPI. Different print sizes correspond to different pixel sizes, which are formulated as follows:

(25) Pix=DPI∗unit

where unit is the actual physical printed size.

**Figure 20:**
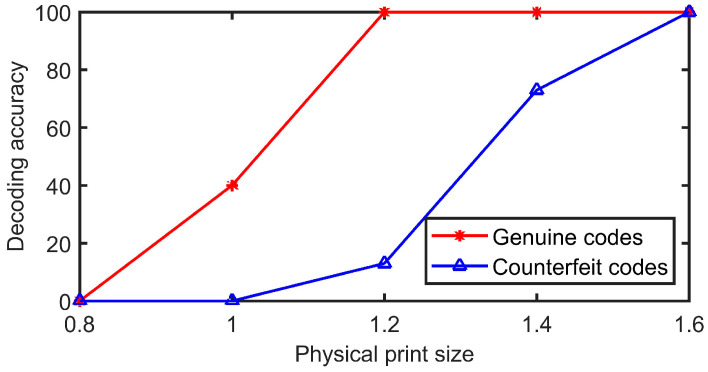
Decoding accuracy of codes of different physical print sizes.

Five codes with print sizes of 0.8 cm, 1 cm, 1.2 cm, 1.5 cm, and 2 cm are generated and printed. Among them, the QR code version and error correction level are 3 and H, and the code point sizes are all 5 × 5. Therefore, the pixel sizes are 348 × 348, 464 × 464, 580 × 580, 696 × 696, and 928 × 928, respectively. After that, the same printer is used to copy and enlarge the copy, resulting in 15 fake codes. The five groups have a total of 25 genuine codes and 75 fake codes. The decoding results are shown in Figure 20, where it can be seen that the decoding accuracy of the genuine codes is not guaranteed when the print size is below 1.2 cm. In contrast, when the print size is larger than 1.5 cm, the decoding accuracy of the counterfeit codes is not guaranteed. Therefore, the print size of 1.2 cm is chosen in this paper to maximize the decoding performance for both genuine and fake products.

### 4.2. Datasets and Evaluation Metrics

#### 4.2.1. Datasets

In this paper, three datasets are constructed: the genuine and counterfeit codes dataset, the attacked codes dataset, and the quality assessment codes dataset. Of these, the first one is used to test the performance of the algorithm, the second one includes genuine codes with different cutting proportions and sizes to test the anti-attacking of the algorithm, and the third one is used to test the performance of the proposed quality assessment algorithm.

(1) Genuine and counterfeit codes dataset

Generate genuine and counterfeit paper codes: a computer generates 10 electronic codes with different semantics, and then prints out 10 paper codes. After that, ten printers of different brands or models, including the official one, are used to make an equal copy. Then, eight printers are used to make 1.5 times and 2 times enlarged copies of the original code, resulting in 260 counterfeit paper codes. Collect sample codes: the portable microscope camera, Anyty, is used to collect 10 genuine paper codes. Collect genuine and counterfeit codes: six mobile phones of different brands or models are used to photograph each paper genuine code five times in different environments. In addition, the mobiles are used to take pictures of each paper counterfeit code one time. The dataset consists of 10 groups, each with a different 16-bit random number for the semantics of the codes. The data of each group includes 1 sample code, 30 genuine codes, and 156 counterfeit codes (among them, 60 are 1 time copies, 48 are 1.5 times copies, and 48 are 2 times copies). The dataset information is shown in Table 1.

(2) Attacked codes dataset

**Figure 21:**
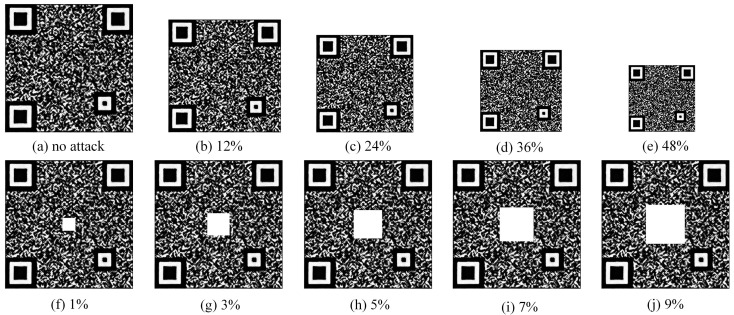
Genuine codes with different reduced proportions and cut ratios ((**a**) has no attack; (**b**–**e**) have cut attack; and (**f**–**j**) have a reduced attack).

Since counterfeit codes will not be recognized as genuine after the attack, only an attack on the genuine codes is performed to check the authenticity algorithm. The size of the 300 genuine codes is reduced in various proportions to 12%, 24%, 36%, and 48% of the original code size. The 300 genuine codes are cut to different degrees, with cut areas of 1%, 3%, 5%, 7%, and 9% of the original code. Figure 21 shows the legend for codes with different reduced proportions and cut ratios. The dataset information is shown in Table 2.

(3) Quality assessment codes dataset

Different degrees of defocus blur (1, 2, 3, 4, and 5) and motion blur (1, 2, 3, 4, and 5) are added to 300 clear digital genuine codes to test the performance of the quality assessment algorithm in this paper. The dataset is presented in Table 3. In the experiments, the printer information used for printing and copying is shown in Table 4, and the smartphone information used for collecting is shown in Table 5.

#### 4.2.2. Evaluation Metrics

To evaluate the performance of the anti-counterfeiting codes and algorithms, three evaluation metrics are formulated as follows:

(26) $$Accuracy=\frac{TP+TN}{TP+TN+FP+FN}$$

(27) $$Precision=\frac{TP}{TP+FP}$$

(28) $$Recall=\frac{TP}{TP+FN}$$

where TP denotes the number of genuine codes judged to be genuine, FN denotes the number of genuine codes judged to be counterfeit, FP denotes the number of counterfeit codes judged to be genuine, and TN denotes the number of counterfeit codes judged to be counterfeit.

For the methods that need to be trained and tested, the following experiments randomly select half of the Table 1 dataset as the training set, however, the testing set is different, which is displayed in the following experiments. The final evaluation metric is the average accuracy of the five experiments randomly combined.

### 4.3. Quality Assessment Algorithm Experiments

The dataset shown in Table 3 is used to validate the effectiveness of the proposed quality assessment algorithm, which is compared with several common algorithms, including Variance, Information Entropy, Tenengrad Gradient, and Fuzzy Probability. The results are shown in Table 6. In this paper, the optimal threshold is chosen by the smaller intra-class variance and the larger inter-class variance. Variance, Information Entropy, and Fuzzy Probability are almost ineffective, and the scores of clear and blurry images are mixed in large numbers. The Tenengrad Gradient can be distinguished to some extent, but there is a large number of crossings. It can be seen that the proposed method outperforms other methods in terms of accuracy, precision, and recall. At the same time, the gap between the other four algorithms is not obvious.

**Figure 22:**
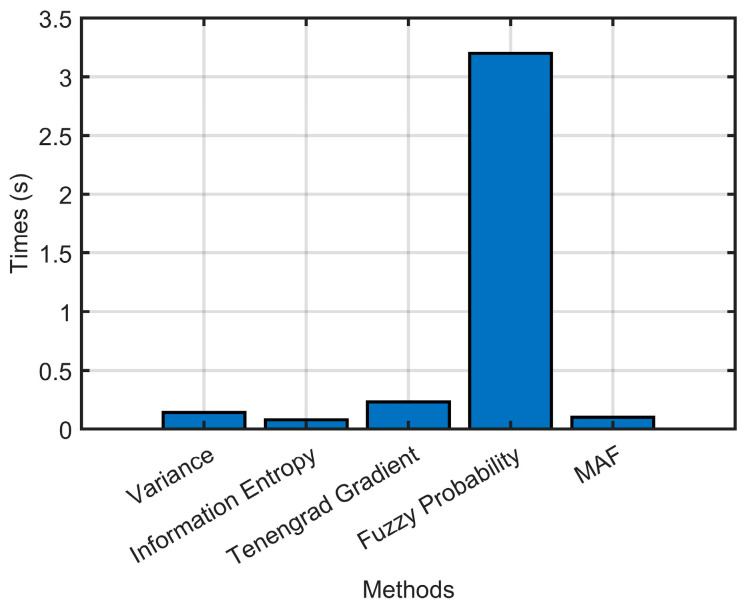
The time consumptions of image quality assessment algorithms for processing one code.

Meanwhile, the quality evaluation method in this paper is able to further distinguish the blur types with 95% accuracy for blurred codes. The specific results are shown in Table 7. The time consumption for processing one code is shown in Figure 22.

### 4.4. Dual Feature Detection Algorithm Experiments

#### 4.4.1. Decodability Analysis

Judging whether the code can be decoded correctly is the first part of the authenticity process which can quickly screen out counterfeit codes. In this paper, the genuine and counterfeit codes dataset (Table 1) is decoded through the QR code decoding program, and the result is shown in Table 8. It can be seen that the genuine codes can be successfully decoded, while the counterfeit codes with an equal ratio have a decoding rate of only 5.83%. However, the enlarged copy codes can reconstruct more details and the decoding rate is significantly improved. The decoding rate is 17.5% for the 1.5 times copy codes and higher for the 2 times copy codes, up to 22.50%. Thus, the genuine code is not necessarily the only one successfully decoded, and the algorithm needs to be further checked to ensure accuracy.

**Figure 23:**
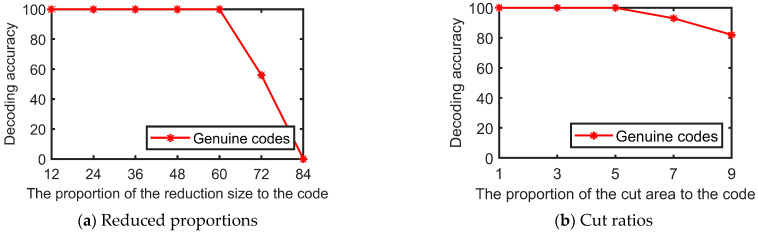
Decoding accuracy of codes on the Table 2 dataset.

QR codes have some error correction capability and up to 30% of the area covered can still be correctly decoded. In this paper, patterns with anti-counterfeiting capability are incorporated into QR codes to ensure some degree of error correction capability. Figure 23a shows the decoding for codes with different reduced proportions. It can be seen that the smaller the proportion, the lower the decoding accuracy. The anti-counterfeiting codes in this paper are resistant to up to 48%. As can be seen in Figure 23b, codes with a maximum cut area of up to 5% can still be guaranteed to decode 100%. In the actual decoding process, the image sizes are also different due to the differences in mobile devices.

#### 4.4.2. DFT Spectral Feature

**Figure 24:**
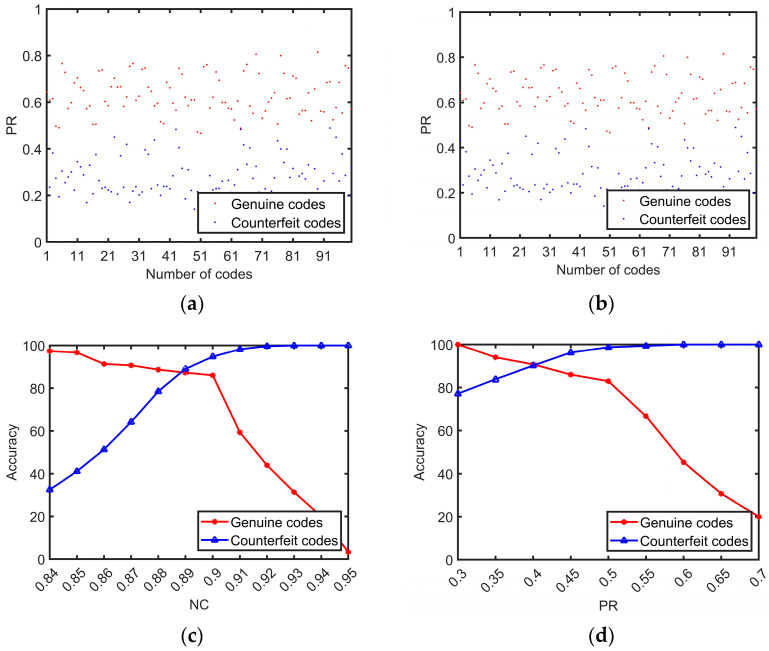
Thresholds of DFT spectral feature ((**a**,**b**) are scatter plots of NC and PR for the two hundred genuine and counterfeit codes; (**c**,**d**) show the accuracies in different thresholds).

The comparison of DFT spectral features is the second part of the discrimination procedure to ensure accuracy. The test consists of two metrics: NC and PR, and a code is identified as genuine only if both metrics reach a threshold. Figure 24a,b shows scatter plots of the two metrics for genuine and counterfeit codes. It can be seen that the distinction between codes is obvious, but there is some crossover in the middle region. To improve the accuracy, the appropriate threshold should be chosen experimentally. Figure 24c,d shows the accuracy results for the genuine and counterfeit codes when the two metrics are chosen with different thresholds. When the NC is in the range of 0.88 to 0.90 and the PR is in the range of 0.4 to 0.5, the accuracy can reach a high level. After selecting the threshold values for the NC and PR, the algorithm is further evaluated, and the result is shown in Table 9. It can be seen that with double feature detection, NC_PR, achieves 100% accuracy, precision, and recall, which is better than using either the NC value or the PR value alone.

#### 4.4.3. Robustness of Dual Feature Detection Algorithm

(1) No attacked test

To further verify the superiority of the proposed dual feature algorithm, the genuine and counterfeit codes dataset shown in Table 1 is used to compare it with several existing popular texture methods and corner methods. For the methods that need to be trained and tested, the following experiments randomly select half of the Table 1 dataset as the testing set. As can be seen in Table 10, the accuracy, precision, and recall ratios of the proposed method all reach 100%. The identification effects of the corner methods (SIFT [34], SURF [35], and BRISK [36]) are relatively general. Some representative texture algorithms (2LQR [24], LBP [37], CLBP [38], CLBC [39] ECLBP [40], COV_LBPD [41], MRELBP [42], JRLP [43], MCDR [44], RALBGC [45], LDEP [46], and LGONBP [47]) can also achieve good results with accuracies above 99%, and some algorithms are close to or reach 100%. In terms of time, to process an image of the same size, the proposed algorithm DFDA needs 0.25 s, which is at the upper level. The shortest time is obtained by [24], only 0.01 s and the longest time is from [46], 146.3 s, followed by [34], 17.08 s. The rest of the methods are within 2 s.

(2) Attacked test

This paper experimentally verifies the robustness of the proposed anti-counterfeiting code and the corresponding algorithm in view of the possible abrasion phenomenon during the use of the code. The dataset in Table 2 is used as the test set. Table 11 shows the accuracy results of the different methods for different reduced proportions. The accuracy of [34,35,36,37,38,39,46,47] and other methods is above 90% when the size is 12%, 24%, or 36% of the original figure. The accuracy of [34,38,39,40,46,47] and other methods is more than 90% when the size of the original image is 64%. When the size is 48% of the original image, only the accuracy of [40] is above 90% (94.39%). The proposed method maintains 100% accuracy during the size ratio reduction from 12% to 48%, which is a significant advantage. Table 12 shows the results of different methods under different cut ratio attacks. The methods in [34,35,36,37,38,42,45] can still maintain more than 90% accuracy under a 1% cut attack, the methods in [34,35,36,45] can still maintain more than 90% accuracy under a 3% cut attack, and the methods in [34,36] can still maintain more than 90% accuracy with a 5% cut attack. The accuracy of the proposed method is 100% during the size reduction from 1% to 5%, which significantly outperforms other methods.

## 5. Conclusions

In this paper, a texture-hidden anti-counterfeiting QR code and a related authentication scheme for mobile devices are proposed. The authentication scheme includes a quality assessment algorithm and dual feature detection. In the test of quality assessment algorithms, the proposed algorithm is compared with several current common algorithms without reference and the superiority of the proposed algorithm is verified. In the authenticity test, the proposed dual feature method is compared with various texture and corner methods. The proposed method achieves an accuracy, precision, and recall of up to 100%, and it also performs well on attacked datasets with reduction and cut. In the following research work, it is necessary to expand the data set (attacks of various physical sizes, higher forgery technologies, and complex application scenarios) to more comprehensively evaluate the practicality of the codes and authentication schemes to achieve convenient pre-sale anti-counterfeit measures.

The dataset used in this paper is obtained by smartphones shooting under indoor conditions, but the collection of 2D codes will also encounter some abnormal conditions, such as insufficient illumination, severe shaking, etc. How to efficiently recover codes and authenticate them is a problem that requires further work. In addition, with multiple forgery devices available, how to authenticate codes using only genuine samples is an important research direction.

## Figures and Tables

**Table 1:** Genuine and counterfeit codes dataset.

	Sample Codes	Genuine Codes	Counterfeit Codes		
			1 Time	1.5 Times	2 Times
Number	10	300	600	480	480

**Table 2:** Attacked codes dataset.

	Reduced Proportions				Cut Ratios				
	12%	24%	36%	48%	1%	3%	5%	7%	9%
Number	300	300	300	300	300	300	300	300	300

**Table 3:** Quality assessment codes dataset.

	Clear	Defocus Blur	Motion Blur
Number	300	1500	1500

**Table 4:** Printer-related brand, model, and DPI information.

Brand	Model	Printing DPI	Replicating DPI
Ricoh Aficio	MP9001	1200 × 1200	600 × 600
Ricoh Aficio	MP6001	1200 × 1200	1200 × 1200
Ricoh Aficio	MP7001	1200 × 1200	600 × 600
Ricoh Aficio	MP9002	1200 × 1200	600 × 600
Ricoh Aficio	Pro907EX	1200 × 1200	600 × 600
Fuji-Xerox	DC-C7550I	2400 × 2400	2400 × 2400
Fuji-Xerox	Apeosport-VC3375	1200 × 2400	1200 × 2400
Fuji-Xerox	Apeosport-IVC5570	1200 × 2400	1200 × 2400
Epson	WF-M21000a	600 × 2400	600 × 600
Epson	L15168	4800 × 2400	600 × 600

**Table 5:** Smartphone-related brand, model, and camera pixel information.

Brand	Model	Camera Pixels
HUAWEI	Mate40 Pro	50 million
HUAWEI	Nova5 Pro	48 million
REDMI	K30	64 million
APPLE	XR	12 million
APPLE	13	12 million
APPLE	8Plus	12 million

**Table 6:** Results of the image quality assessment algorithms on the Table 3 dataset.

Methods	Intra-Class Variance	Inter-Class Variance	Accuracy (%)	Precision (%)	Recall (%)
Variance	78.04	10.67	72.67	56.72	76
Information Entropy	0.06	0.01	57.67	41.40	65
Tenengrad Gradient	11.52	17.44	88.33	75.60	96
Fuzzy Probability	0.01	0.01	76.67	62.30	76
**MAF**	**512.91**	**1099.39**	**96**	**91.80**	**97**

**Table 7:** Results of blur types.

Method	The Number of Defocus Blur Correctly Determined	The Number of Motion Blur Correctly Determined	Accuracy (%)
MAF	91	97	95

**Table 8:** Decoding accuracy of codes on the Table 1 dataset.

Codes	Total Number	Decoding Number	Decoding Rate (%)
Genuine	300	300	100
1 time copy	600	35	5.83
1.5 times copy	480	84	17.50
2 times copy	480	108	22.50

**Table 9:** Accuracy, precision, and recall of the DFT spectral feature on the Table 1 dataset.

Methods	Accuracy (%)	Precision (%)	Recall (%)
NC	98.78	87.88	100
PR	93.42	59.54	79.31
**NC_PR**	**100**	**100**	**100**

**Table 10:** Accuracy ratios, precision ratios, recall ratios, and time of different methods from the Table 1 dataset.

Methods	Accuracy (%)	Precision (%)	Recall (%)	Time (s)
2LQR [24]	24.05	6.32	80.54	0.01
SIFT [34]	97.30	91.25	98.65	17.08
SURF [35]	95.37	87.76	57.33	1.62
BRISK [36]	97.28	87.61	76.74	0.46
LBP [37]	99.25	100	88.89	0.09
CLBP [38]	99.54	93.10	100	0.18
CLBC [39]	99.79	96.42	100	0.17
ECLBP [40]	99.50	98.08	94.44	0.17
COV_LBPD [41]	99.50	98.43	100	1.33
MRELBP [42]	100	100	100	1.64
JRLP [43]	100	100	100	0.61
MCDR [44]	99.88	100	98.15	1.75
RALBGC [45]	99.87	100	98.23	1.31
LDEP [46]	100	100	100	146.3
LGONBP [47]	99.88	100	98.15	1.45
**DFDA**	**100**	**100**	**100**	0.25

**Table 11:** Recall ratios of different methods on the Table 2 reduction dataset.

Methods	12%	24%	36%	48%
2LQR [24]	78.24	76.29	73.15	70.54
SIFT [34]	95.83	95.83	91.66	87.37
SURF [35]	95.12	90.33	89.56	0
BRISK [36]	97.02	91.45	76.23	0
LBP [37]	56.07	42.99	57.00	37.38
CLBP [38]	100	99.06	96.26	34.57
CLBC [39]	100	100	96.26	33.64
ECLBP [40]	98.13	99.06	99.06	94.39
COV_LBPD [41]	39.71	29.90	28.97	21.02
MRELBP [42]	62.61	62.61	40.18	39.53
JRLP [43]	7.47	5.75	0	0
MCDR [44]	34.57	0	0	0
RALBGC [45]	80.37	70.09	66.63	50.46
LDEP [46]	100	100	99.06	69.19
LGONBP [47]	92.52	90.34	90.05	84.56
**DFDA**	**100**	**100**	**100**	**100**

**Table 12:** Recall ratios of different methods on the Table 2 cut dataset.

Methods	1%	3%	5%	7%	9%
2LQR [24]	79.15	75.27	66.81	56.24	48.71
SIFT [34]	95.64	93.64	90.64	89.64	85.64
SURF [35]	92.67	91.45	88.76	80.70	76
BRISK [36]	93.25	92.25	92.05	75.25	23.25
LBP [37]	58.87	42.05	40.33	15.21	10.23
CLBP [38]	93.45	83.17	66.35	16.82	14.68
CLBC [39]	92.52	77.57	46.72	38.31	16.82
ECLBP [40]	21.49	7.47	5.60	3.73	2.80
COV_LBPD [41]	34.57	18.22	19.62	17.75	16.35
MRELBP [42]	91.58	82.54	79.43	73.83	66.35
JRLP [43]	0	0	0	0	0
MCDR [44]	0	0	0	0	0
RALBGC [45]	98.13	91.58	89.98	77.57	75.70
LDEP [46]	85.04	81.30	54.20	28.03	11.21
LGONBP [47]	89.71	88.45	87.85	66.35	26.26
**DFDA**	**100**	**100**	**100**	86.56	75.32

## Data Availability

Not applicable.
